# Physiology of endothelin in producing myocardial perfusion heterogeneity: A mechanistic study using darusentan and positron emission tomography

**DOI:** 10.1007/s12350-013-9756-5

**Published:** 2013-07-11

**Authors:** Nils P. Johnson, K. Lance Gould

**Affiliations:** Weatherhead PET Center For Preventing and Reversing Atherosclerosis, Division of Cardiology, Department of Medicine, University of Texas Medical School and Memorial Hermann Hospital, 6431 Fannin St., Room 4.256 MSB, Houston, TX 77030 USA

**Keywords:** Endothelial dysfunction, PET imaging, coronary blood flow

## Abstract

**Background:**

Heterogeneity of resting perfusion may be due in part to up-regulation of coronary vasoconstriction via endothelin (ET) type A receptors, as homogeneity increases during subsequent vasodilatory hyperemia. Therefore, we conducted a mechanistic study using an ET receptor antagonist to determine if it could alter the homogeneity of myocardial perfusion.

**Methods:**

Included subjects demonstrated a low myocardial perfusion homogeneity index (HI) compared to normal volunteers. Four serial cardiac positron emission tomography Rb-82 scans were performed 2 weeks apart. Before the middle two scans, subjects were randomized to receive either darusentan first then placebo or visa versa. Absolute flow and coronary flow reserve were quantified for each study. Rest flow was adjusted for the pressure-rate product (PRP).

**Results:**

We screened 37 subjects and randomized 20 who satisfied entry criteria. Rest HI increased significantly while taking darusentan (0.39 ± 0.10 vs 0.33 ± 0.04 on placebo, *P* = .030, compared to a normal range of 0.52 ± 0.10) without an increase in the PRP (6,859 ± 1,503 vs 6,976 ± 1,092, *P* = .79), leading to a higher adjusted flow at rest (0.69 ± 0.18 cc/minute/g at 7,000 PRP vs 0.59 ± 0.07 with placebo).

**Conclusions:**

Antagonism of the type A ET receptor increases homogeneity of resting myocardial perfusion. The mechanism appears to be increased absolute rest flow without an increase in either the PRP or myocardial perfusion during hyperemia. Our translational results are consistent with one mechanism for the observed heterogeneity of myocardial perfusion in humans.

## Introduction

Myocardial perfusion never appears completely homogeneous, whether studied on a smaller scale by microsphere injections^1^ or on a larger scale by cardiac positron emission tomography (PET) imaging.^2^ Heterogeneity of myocardial perfusion may occur for a variety of reasons, including the fundamental fractal vascular structure of the coronary circulation,^1^ time-dependent capillary “twinkling” as shown by microscopy,^3^ imaging technique (for example, count statistics or smoothing filters), and alterations in coronary vasomotor tone. Endothelin (ET) plays a key role in this last mechanism, as its action on type A receptors produces vasoconstriction, serving as one counter mechanism to the effects of nitric oxide and other endogenous vasodilators.^4^

Prior animal work from our group has demonstrated that adenosine can variably reverse perfusion defects produced by intracoronary infusion of the major vascular isoform ET-1.^5^ Such a mechanism may partially account for our observation that heterogeneity of resting myocardial perfusion, and its improvement after dipyridamole, predict even mild stress-induced abnormalities.^2^ Homogeneity of myocardial perfusion associates inversely with classic atherosclerotic risk factors and disease burden.^6^ Even in young, normal volunteers, occult risk factors significantly reduce perfusion homogeneity.^7^

Pharmacologic antagonism of ET in humans has become possible with a variety of agents. However, their effect on myocardial perfusion homogeneity has not been studied. Therefore, we performed cardiac PET before and after ET blockade with darusentan, an oral antagonist of the ET type A receptor. Our primary hypothesis was that darusentan would increase homogeneity of myocardial perfusion at rest, thereby demonstrating one mechanism for perfusion heterogeneity. Secondary aims were to assess the reproducibility of perfusion homogeneity, the duration of perfusion changes after darusentan administration, and if its effects on heterogeneity were mediated by altering absolute myocardial flow and coronary flow reserve (CFR).

## Methods

### Study Design

Our study utilized an investigator-initiated, single center, crossover, placebo-controlled, blinded trial with 1:1 randomization. It was performed at the Weatherhead PET Center for Preventing and Reversing Atherosclerosis of the University of Texas Medical School at Houston and Memorial Hermann Hospital. Each subject gave written informed consent approved by the institutional review board.

Subjects underwent four sequential cardiac PET scans: baseline, placebo, darusentan, and final. Placebo and darusentan scans occurred second and third, ordered randomly when study eligibility had been confirmed from the baseline labs and PET. Drug administration had to occur within 4 weeks of the baseline PET scan, although all cases started the next day except 1 subject (3 weeks). Each drug was taken for 14 ± 4 days. Fasting blood work (complete blood count, comprehensive chemistry panel, serum uric acid, phosphorous, lactate dehydrogenase, and γ-glutamyl transpeptidase) and urinalysis were performed at most 4 weeks before the baseline PET and on the day of the final PET. A lipid profile was performed at baseline.

**Figure 1:**
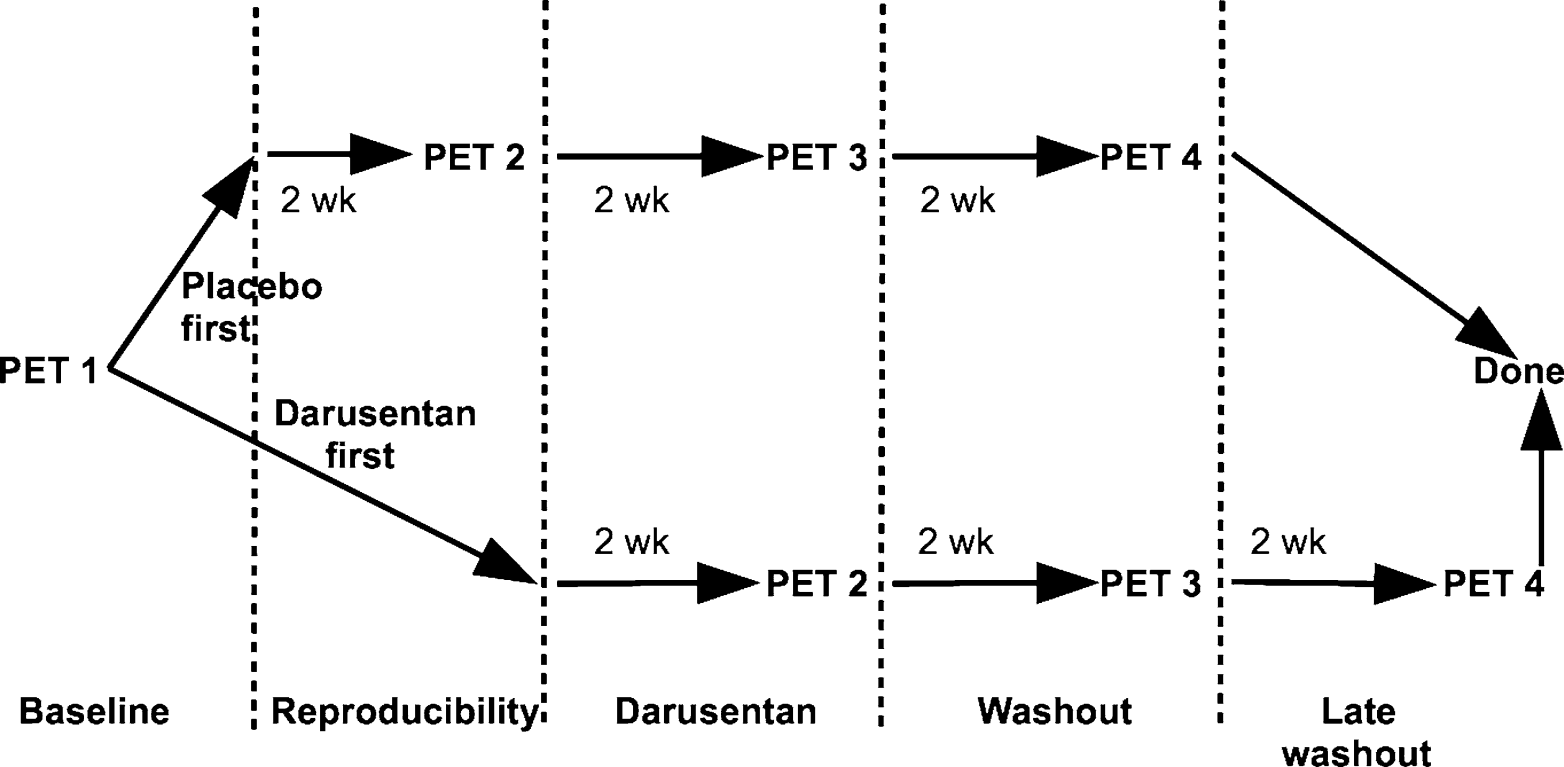
Study design. If all entry criteria were met after the baseline PET scan, subjects were randomized to receive either placebo or darusentan first, before crossing over to receive the other treatment. Four sequential PET scans (#1 to #4) were performed after every 2-week interval. Each subject had a baseline, darusentan, and washout PET study. Based on the order determined by randomization, subjects had a PET study to assess either reproducibility or late washout but not both

Randomization of the placebo and darusentan order allowed for assessment as follows. Subjects whose placebo scan immediately followed the baseline scan provided information on the test-retest reproducibility of myocardial homogeneity. Subjects whose placebo scan instead followed the darusentan scan provided information on late washout. However, all subjects had baseline, darusentan, and washout studies. Figure 1 depicts the study schematic.

### Subject Recruitment and Entry Criteria

Subjects at least 18 years or older were recruited from our database of prior PET scans. Inclusion criterion was a rest myocardial heterogeneity at least 2 standard deviations below originally published normal limits based on an older, rotating rod PET scanner used at that time.^2^ During the study, this criterion was relaxed to at least 1.5 standard deviations to increase enrollment. See the next section for explicit cutoffs based on our measure of homogeneity. Exclusion criteria broadly included uncontrolled or severe comorbidity, inability to undergo serial PET scans, or contraindication to darusentan.

Specific exclusion criteria were: acute heart failure, sustained and symptomatic hypotension (systolic <90 mm Hg), uncontrolled hypertension (systolic ≥170 mm Hg or diastolic ≥100 mm Hg), unstable angina, major adverse cardiac event in prior 6 months (acute myocardial infarction, stroke, transient ischemic attack, or mechanical revascularization), primary valvular disease, significant vascular aneurysm, history of renal or liver failure (elevated serum creatinine, total bilirubin ≥3 mg/dL, or serum liver aminotransferase enzyme levels above twofold the upper limit of normal), active malignancy, life expectancy under 12 months, uncontrolled diabetes mellitus or complications of gastroparesis or severe neuropathy, substance abuse within the last 2 years, active or recent participation within 1 month of enrollment in another clinical research study, planned surgical procedure during study, certain herbal food supplements (l-carnitine, l-arginine, Ginko biloba), prior serious adverse event attributed to treatment with any ET receptor agonist, or female subjects who are pregnant or with the potential for child-bearing, lactating, or treated with hormone therapies.

Potential subjects who had undergone prior PET studies were contacted by mailings after a preliminary chart review. Those who were interested signed informed consent and underwent fasting blood work in advance of the baseline PET scan. If laboratory testing and medical review did not identify any exclusion criteria, then the baseline PET scan was performed to determine the resting perfusion homogeneity. Only subjects who met the baseline inclusion criterion underwent randomization.

Assignment of subjects to study drug order (placebo then darusentan or darusentan then placebo) was performed by the dedicated research pharmacy at our institution using simple 1:1 randomization. An allocation sequence was created using a random number generator by a statistician not associated with the study. A printed list of the allocation sequences was concealed in the research pharmacy and used sequentially for each randomized subject. Investigators, study subjects, and PET center staff performing imaging were blinded to treatment assignment during the entire trial.

Medications were dispensed in pill bottles containing either 18 tablets of darusentan 100 mg or identical placebo. Each subject was given one bottle during a treatment period and returned unused pills on the day of the associated PET scan. Pill counts performed by the research pharmacy assessed compliance and found no significant deviations. Subjects were instructed to take one pill each morning including on the day of a PET scan. Although no study drug intolerance was identified, the protocol mandated withdrawal of any subject unable to take the study drug, as dose reduction was not permitted. During the study, subjects were instructed to continue all concomitant medications consistently. At each PET scan, subjects reported on any symptoms noted while taking the study medication. Symptoms were classified without knowledge of treatment assignment as headache, congestion, flushing, and other (including chest tightness, sore throat, edema, myalgia, nervousness, lightheadedness).

A required sample size of about 40 subjects was estimated based on a paired *t* test with *α* = 0.05, *β* = 0.80, and a projected increase from 0.37 to 0.43, which is approximately half a standard deviation of the normal range for our previously published^2^ index of myocardial homogeneity (described next). No interim analyses or stopping guidelines were considered.

### Homogeneity Index (HI)

We previously developed the unitless HI using Markovian analysis of relative uptake images.^2^ In brief, the HI quantifies the intuitive, visual notion of homogeneity. Values near HI = 1 occur when relative uptake is completely uniform. Values near HI = 0 occur when every uptake pixel differs from its neighbors. To avoid quantifying heterogeneity in areas of scar or normal myocardium, uptake values below 50% or above 85% of maximum are set to 50% or 85%, respectively. By design, the HI favors small uptake differences among adjacent pixels, for example those present at interfaces among normal myocardium, scar, and perfusion defects. Four basal slices were not used for HI analysis due to low counts in the membranous interventricular septum. Two apical slices were not used for HI analysis due to potential partial volume errors caused by partial thickness slices through the LV apex and apical motion.

**Figure 2:**
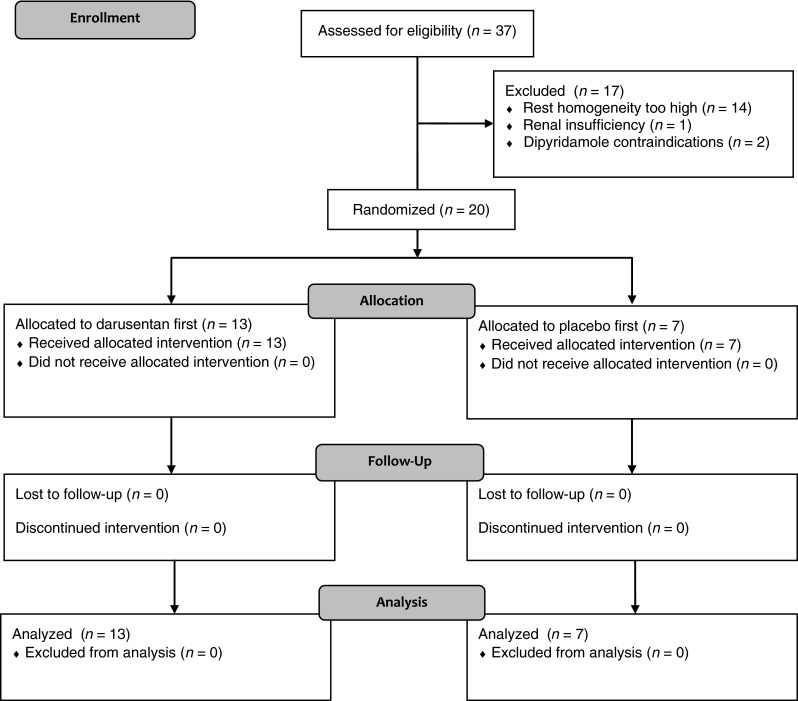
Enrollment flow diagram. CONSORT flow diagram of study enrollment, allocation, follow-up, and analysis

Our original development of the HI described normal limits at rest of 0.63 ± 0.13 based on PET images from prior generation rotating rod PET scanner.^2^ However, during the course of this trial, we carried out a more rigorous study of young, asymptomatic, normal volunteers using a higher resolution PET scanner with computed tomography (CT) attenuation correction to determine “true normal” limits of 0.52 ± 0.10 with the same scanner used for the clinical trial.^7^

Based on a rest cutoff of at least 2 standard deviations below originally published normal limits,^2^ we originally required subjects to have rest HI ≤ 0.63 − (2 × 0.13) = 0.37. To increase enrollment, the cutoff was relaxed to a rest HI ≤ 0.63 − (1.5 × 0.13) = 0.44 after the first 21 subjects had been screened and the first 9 had been randomized. However, only 4 (20%) of the 20 randomized subjects had a rest HI > 0.37. Due to slight asymmetry of the HI distribution in normal volunteers imaged by PET-CT, only 2 (10%) of the 20 randomized subjects had an entry rest HI that would not be considered an outlier using box-and-whisker plots, as detailed in a subsequent figure.

### PET Acquisition and Reconstruction Protocol

Our imaging protocol has been described previously.^7^ In brief, subjects were instructed to fast for 4 hours and abstain from caffeine, theophylline, and cigarettes for 24 hours. Cardiac PET was performed using a Discovery ST 16-slice PET-CT machine (GE Healthcare) in two-dimensional mode with settings for an in-plane resolution of approximately 6-7 mm full-width at half-maximum.

Rest emission images were obtained over 7 minutes beginning immediately upon intravenous injection of 1,110 to 1,850 MBq (30 to 50 mCi) of generator-produced Rb-82 (Bracco Diagnostics). The first 2 minutes of emission images were binned to form arterial input data. The last 5 minutes of emission images were binned to form myocardial uptake data. Immediately after completion of the resting scan, dipyridamole (142 mcg/kg/minute) was infused for 4 minutes. Four minutes after completion of dipyridamole infusion, the same dose of radiotracer was given. Stress emission images were acquired for 7 minutes and binned into arterial and myocardial images as for the resting scan. Dipyridamole-induced symptoms were treated with intravenous aminophylline.

CT scans for attenuation correction were acquired before rest and after stress emission imaging. Protocols for attenuation CT acquisition have been previously reported.^8^ Fusion images superimposed PET emission and CT transmission scans in horizontal, coronal, and sagittal views. Co-registration was optimized by shifting as needed. PET images were reconstructed using filtered backprojection with a Butterworth filter (cutoff 0.55 cycles/cm, rolloff 10 dB/decade, pixel size 3.27 × 3.27 mm). After attenuation correction and reconstruction, transaxial PET images were exported for analysis on CARDIAC software (Positron Corporation) to generate true short- and long-axis views, perpendicular and parallel to the long-axis of the LV. Circumferential profiles of maximum radial activity for each true short-axis slice were used to construct two-dimensional topographic views of the entire LV in lateral, inferior, septal, and anterior quadrant views.

In one case, the GE scanner required maintenance and the subject could not reschedule for logistic reasons. Instead, an mPower rotating rod PET scanner (Positron Corporation) was used for imaging. In another case, the GE scanner suffered a technical malfunction during stress acquisition so that only rest data was available. For the last subject, the Rb-82 generator was recalled after the third PET scan. In this case, the final PET scan took place 29 days later using N-13 ammonia.

### Primary and Secondary Endpoints

The primary endpoint was the change in rest myocardial perfusion homogeneity as quantified by the unitless HI. Calculation of HI was completely automated by software and occurred on the day of the PET scan, without subsequent reprocessing or alteration.

Secondary endpoints were stress HI (computed as for rest HI but on the stress uptake images), absolute myocardial flow, and CFR. Originally planned secondary endpoints of relative uptake perfusion defects and the base-to-apex longitudinal gradient^9^ were modified since we developed the technical ability to measure absolute flow routinely. Peak integrated activity over an approximate 2 × 2 mm circular area in the left atrium or thoracic aorta was determined from transaxial images acquired during the first 2 minutes after each radiotracer injection. Integrated myocardial activity during the next 5 minutes was determined from topographical maps of the LV. For each of 64 radial segments at each of 21 short-axis slices, integrated arterial input and myocardial uptake were used to compute absolute myocardial flow using an established model^10^ implemented using custom software. The 21 × 64 pixel topographic flow map was smoothed using a 5 × 5 pixel average to suppress noise introduced by the flow model. CFR was computed as the stress-to-rest ratio on a pixel-by-pixel basis. Rest flow was adjusted for baseline pressure-rate product (PRP) by dividing its measured value by the systolic blood pressure and heart rate, then multiplying by 7,000 bpm mm Hg (approximately the average resting PRP at baseline).

### Statistical Methods

Statistical analysis was performed using R version 2.13.1 (R Foundation for Statistical Computing, Vienna, Austria). Continuous variables are expressed as mean ± standard deviation. Frequency variables are expressed as number (percent). All continuous variables were found to be approximately normal on inspection of quantile-quantile plots. ANOVA from a linear mixed effects model with random effects within subjects assessed changes in primary and secondary endpoints among baseline, darusentan, and washout PET scans. If this ANOVA was significant, then a Tukey all-pair comparison was performed to determine which PET scan(s) were different. A paired *t* test compared reproducibility to baseline (for subjects randomized to placebo first), late image to washout (for subjects randomized to darusentan first), and baseline and final laboratory values (for all patients). McNemar’s *χ* ^2^ test compared paired symptoms between placebo and darusentan. Correlation between continuous variables used the Pearson coefficient. Bland-Altman analysis used a standard 95% confidence interval for the limits of agreement. Logistic regression models were used in a post hoc search for predictors of darusentan response. All applicable tests were two-tailed and *P* < .05 was considered statistically significant. Box plots identify outliers as 1.5 times the interquartile range.

### Role of the Academic Authors and Manufacturer

Gilead Sciences supported the trial by supplying the darusentan and placebo tablets. The corresponding author obtained an investigator-initiated Investigational New Drug (IND) permit for darusentan. Gilead paid our academic medical center an option fee for exclusive use of any technology stemming from the study. Although Gilead offered to pay for serum and urine laboratory studies, this option was not exercised and all laboratory and PET studies were paid for via internal funding from the Weatherhead PET Center for Preventing and Reversing Atherosclerosis. The academic authors had full access to the data, carried out its analysis, and wrote the manuscript independent of the manufacturer. However, Gilead did review the manuscript for confidential or proprietary information but requested no changes. The trial was registered with clinicaltrials.gov (identifier NCT00738049).

## Results

Subjects underwent enrollment between May 2009 and July 2011. The trial was stopped after 20 enrollments as the manufacturer of darusentan (Gilead Sciences) halted further development of the drug after a negative phase 3 trial in resistant hypertension.^11^ Figure 2 shows the flow diagram for the trial. All subjects underwent all 4 PET scans and received all intended treatments, with no losses or exclusions after randomization. Table 1 lists the baseline and clinical characteristics of the participants. No adverse events occurred during the study. Symptoms were reported by 6 (30%) subjects taking placebo (3 headache, 1 flushing, 0 congestion, 3 other) but 16 (80%) subjects taking darusentan (9 headache, 4 flushing, 4 congestion, 4 other), which differed significantly (*P* = .009).

**Table 1:** Baseline characteristics

Number of subjects	20
Age (years)	61 ± 12
Male	19 (95%)
Weight (lbs)	208 ± 26
Body mass index (kg/m^2^)	29.2 ± 3.6
Hypertension	11 (55%)
Family history of early atherosclerosis	6 (30%)
Diabetes mellitus	0 (0%)
Current or prior tobacco	6 (30%)
Dyslipidemia	20 (100%)
Prior mechanical revascularization*	1 (5%)
Prior myocardial infarction*	1 (5%)
Baseline coronary calcium	15 (75%)
Aspirin	14 (70%)
Statin	18 (90%)
Niacin	7 (35%)
Fibrate	3 (15%)
Ezetimibe	2 (10%)
Beta blocker	9 (45%)
Angiotensin blocker	7 (35%)
Total cholesterol (mg/dL)	154 ± 32
HDL (mg/dL)	56 ± 15
LDL (mg/dL)	78 ± 29
Triglycerides (mg/dL)	97 ± 42
Non-HDL cholesterol (mg/dL)	98 ± 34

* Same subject had prior myocardial infarction and mechanical revascularization

**Figure 3:**
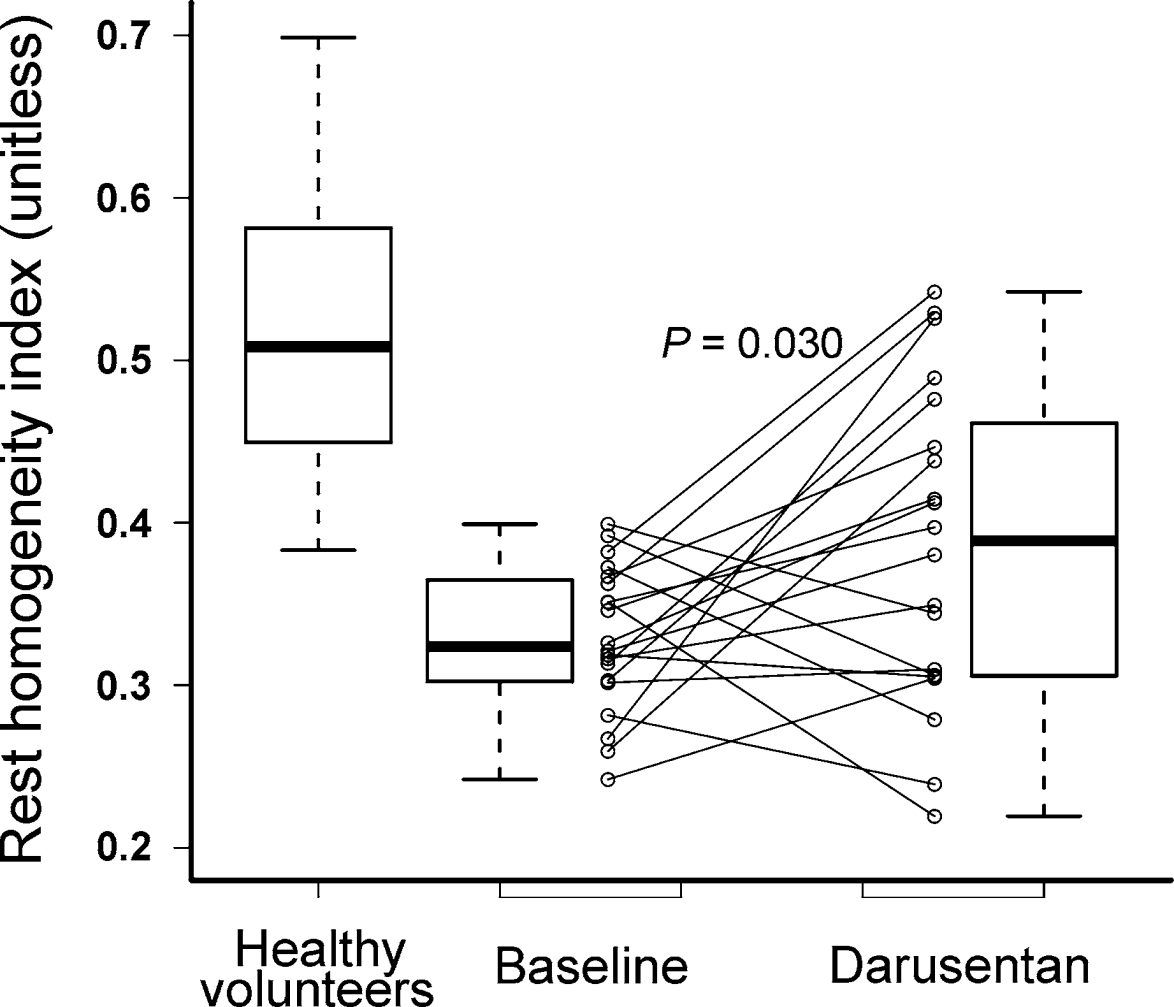
Paired homogeneity index. Myocardial perfusion homogeneity under resting conditions significantly increased during darusentan treatment compared to baseline (*P* = .030). Typical values of resting homogeneity from 56 young, asymptomatic “true normal” volunteers come from our prior work.^7^ Paired homogeneity index values for all 20 study subjects are shown in addition to summary box plots

**Figure 4:**
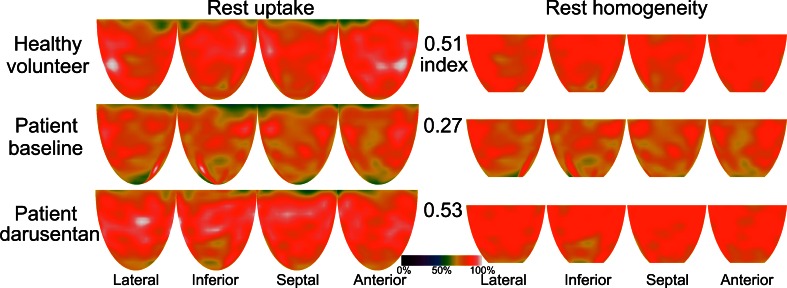
Example images. Relative uptake maps of the four LV quadrants are shown for a normal volunteer (*top row*) from our prior work^7^ as well as a notable patient at baseline (*middle row*) and while taking darusentan (*bottom row*). The *left column* shows the raw uptake images while the *right column* displays them as analyzed for homogeneity (range truncated to 50%-85%, basal four and apical two slices removed) along with the homogeneity index value. Whole LV average rest flow increased from 0.51 cc/minute/g at 7,000 bpm mm Hg at baseline to 0.74 with darusentan

Rest HI differed significantly among scans (ANOVA from linear mixed effects model *P* value = .030) and was significantly higher (Tukey *P* value = .019) while taking darusentan (0.39 ± 0.10) compared to baseline (0.33 ± 0.04). Figure 3 shows baseline and darusentan values for the rest HI, including data on 56 “true normals” previously published from young, asymptomatic volunteers who underwent screening for occult factors before PET perfusion imaging using the same scanner as in this darusentan trial.^7^ Table 2 lists HI, hemodynamics, and absolute flow and CFR for the baseline, darusentan, and washout PET scans. Average rest flow when adjusted for PRP differed significantly among scans (ANOVA from linear mixed effects model *P* value = .012) and was significantly higher (Tukey *P* value = .005) while taking darusentan (0.69 ± 0.18 cc/minute/g at 7,000 mm Hg bpm) compared to baseline (0.59 ± 0.07 cc/minute/g at 7,000 mm Hg bpm). Figure 4 depicts rest images in a normal volunteer and in a study patient at baseline and while taking darusentan.

**Table 2:** Primary and secondary endpoints

	Baseline	Darusentan	Washout	*P* value
Rest homogeneity (unitless)	0.33 ± 0.04	0.39 ± 0.10*	0.37 ± 0.08	.030
Stress homogeneity (unitless)	0.45 ± 0.08	0.43 ± 0.10	0.43 ± 0.10	.90
Stress-rest homogeneity change (unitless)	0.12 ± 0.09	0.04 ± 0.09	0.06 ± 0.10	.13
Rest systolic blood pressure (mm Hg)	116 ± 12	107 ± 13*	115 ± 14^†^	<.001
Rest diastolic blood pressure (mm Hg)	66 ± 9	60 ± 9*	66 ± 9^†^	<.001
Rest heart rate (bpm)	60 ± 7	64 ± 9*	59 ± 9^†^	.008
Rest pressure-rate product (bpm mm Hg)	6,976 ± 1,092	6,859 ± 1,503	6,817 ± 1,382	.79
Stress systolic blood pressure (mm Hg)	116 ± 11	114 ± 14	117 ± 13	.26
Stress diastolic blood pressure (mm Hg)	64 ± 9	60 ± 8*	65 ± 10^†^	.001
Stress heart rate (bpm)	85 ± 10	87 ± 10	84 ± 11	.32
Rest Rb-82 dose (mCi)	33.0 ± 5.0	32.5 ± 4.6	31.6 ± 6.5	.69
Stress Rb-82 dose (mCi)	32.9 ± 5.0	32.5 ± 4.6	31.7 ± 6.1	.73
Rest average flow (cc/minute/g)	0.58 ± 0.10	0.65 ± 0.13	0.60 ± 0.11	.09
PRP-adjusted rest average flow (cc/minute/g at 7,000 bpm mm Hg)	0.59 ± 0.07	0.69 ± 0.18*	0.63 ± 0.14	.012
Stress average flow (cc/minute/g)	2.03 ± 0.56	1.97 ± 0.54	1.83 ± 0.55	.30
CFR average (unitless)	3.52 ± 0.80	3.04 ± 0.61	3.11 ± 0.77	.06

* *P* < .05 compared to baseline

^†^ *P* < .05 compared to darusentan

A significant correlation existed between rest PRP and average absolute flow at baseline (*r* = 0.71, 95% confidence interval 0.39 to 0.88, *P* < .001), as expected and previously described.^6^ However, the correlation between rest PRP and average flow while taking daursentan was not significant (*r* = 0.25, 95% confidence interval −0.21 to 0.63, *P* = .28), suggesting an uncoupling of PRP from absolute flow as seen with other vasodilators such as dipyridamole.^12^

Retest values of rest HI (0.39 ± 0.12) did not differ significantly from baseline (0.33 ± 0.04, paired *t* test *P* = .26). Bland-Altman analysis of baseline and reproducibility rest HI showed a mean difference (reproducibility minus baseline) of +0.05 (95% confidence interval −0.16 to +0.26). Late washout values of rest HI (0.35 ± 0.06) did not decrease further from washout (0.37 ± 0.08, paired *t* test *P* = .66). Bland-Altman analysis of washout and late washout showed a mean difference (late minus washout) of −0.01 (95% confidence interval −0.17 to +0.15). Rest HI did not differ significantly between washout and baseline (paired *t* test *P* = .14).

As can be seen in Figure 3, there were 13 (65%) subjects whose rest HI on darusentan increased by ≥10%, 5 (25%) whose rest HI decreased by ≥10%, and 2 (10%) subjects whose rest HI remained within 10% of its baseline value. In a post hoc analysis, several variables (darusentan-induced side effects, age, body mass index, coronary calcium, hypertension, family history of early atherosclerosis, prior tobacco, LDL cholesterol, non-HDL cholesterol, baseline HI value, baseline blood pressure, and change in systolic blood pressure, diastolic blood pressure, heart rate, and absolute rest flow from baseline when taking darusentan) were examined as predictors for rest HI response to darusentan (binary increase or decrease). Only body mass index (*P* = .046) and change in rest flow (*P* = .056) had *P* < .10, and when studied together neither remained a significant predictor of rest HI response to active treatment (adjusted *P* = .55 and *P* = .30, respectively).

Laboratory values did not differ clinically between the baseline and final PET scans, as shown in Table 3. No significant correlation existed between rest HI and body mass index (*r* ^2^ = 0.05, *P* = .37) or injected Rb-82 dose (*r* ^2^ < 0.01, *P* = .60).

**Table 3:** Laboratory values

Parameter	Baseline	Final	*P* value
Glucose (mg/dL)	85 ± 14	81 ± 15	.28
Blood urea nitrogen (mg/dL)	18 ± 5	19 ± 6	.14
Creatinine (mg/dL)	1.1 ± 0.2	1.0 ± 0.2	.11
Potassium (mmol/L)	4.6 ± 0.5	4.7 ± 0.5	.45
Total bilirubin (mg/dL)	0.8 ± 0.3	0.7 ± 0.3	.70
AST (U/L)	27 ± 11	26 ± 6	.58
ALT (U/L)	27 ± 10	26 ± 6	.48
Phosphorous (mg/dL)	3.3 ± 0.4	3.5 ± 0.5	.026
Uric acid (mg/dL)	6.6 ± 1.3	6.7 ± 1.5	.69
Lactate dehydrogenase (U/L)	177 ± 53	181 ± 65	.82
γ-Glutamyl transpeptidase (U/L)	30 ± 29	30 ± 26	.82
White blood cell count (1,000/μL)	6.5 ± 1.3	6.5 ± 1.6	.98
Hemoglobin (g/dL)	15.0 ± 0.9	14.7 ± 0.8	.034
Platelet count (1,000/μL)	194 ± 39	195 ± 44	.97
Urinalysis (not normal)	1 (5%)	1 (5%)	1.00

## Discussion

Our translational results support a mechanistic role for ET in regulating perfusion homogeneity in the coronary circulation. To our knowledge, we are the first to modulate ET directly in humans for the purpose of studying myocardial perfusion homogeneity. Treatment with the ET antagonist darusentan significantly improved resting myocardial perfusion homogeneity.

Improved perfusion homogeneity with darusentan occurred as average absolute resting flow increased, especially when accounting for a slightly lower PRP. As darusentan has been shown to lower blood pressure,^13^ the observed decrease in these parameters during its administration is expected and consistent. Normally, however, PRP and resting flow are directly related. Therefore, the flat or even inverse relationship between PRP and resting flow in this data suggests that ET blockade increases flow by an alternative mechanism, likely vasodilation.

Note that if ET blockade raised all flows by the same amount, homogeneity would remain largely unchanged as it depends on relative differences. Only altering flow differentially will decrease heterogeneity, as variation among regions diminishes. Thus, improved homogeneity with darusentan provides an important physiologic insight distinct from the obvious and known vasodilation known to occur with ET antagonism.

Our HI was reproducible under test-retest conditions, which supports its use as an imaging biomarker. Darusentan did not produce any lasting clinical changes in rest perfusion homogeneity, blood pressure, or laboratory values beyond the study period. Late washout values of HI did not differ from those obtained after a typical washout period, consistent with the known biologic 12.5 hour half-life of darusentan.

While darusentan improves rest HI, it does not reach levels seen in young, normal volunteers, suggesting residual factors that produce heterogeneity. This improved but persistent heterogeneity after darusentan parallels our prior animal study in which *intravenous* adenosine improved but did not normalize myocardial perfusion defects produced by intracoronary ET-1, whereas *intracoronary* adenosine eliminated the residual perfusion images remaining after intravenous adenosine.^5^ Higher doses of darusentan (up to 300 mg can be given daily) may have produced additional increases in perfusion homogeneity beyond those seen by our 100 mg dose. Additionally, permanent structural changes in the myocardium may have developed due to risk factors or manifest heart disease that prevent normalization of rest HI.

In a minority of subjects, the rest HI on darusentan either did not increase or even decreased. No predictors of this response were found in a post hoc analysis. By analogy, about half of subjects with resistant hypertension randomized to 100 or 300 mg did not reach blood pressure goals.^13^ Also, certain polymorphisms of the mediating G-protein beta3 subunit may modulate the vasoconstrictor effect of the ET system,^14^ and therefore be important determinants of treatment response to darusentan. Future work should examine the subgroup of nonresponders for additional mechanistic insights.

Some potential contributors to perfusion heterogeneity arise from the imaging technique: Poisson counting statistics inherent to radioisotopes, scatter and attenuation of photons, PET camera noise, selected reconstruction algorithm, and the applied smoothing filter. Our demonstrated lack of correlation between the rest HI and injected dose or body mass index, as well as good test-retest reproducibility, suggest that these mechanisms are not dominant.

### Comparison to Existing Literature

To our knowledge, perfusion heterogeneity in humans has not been studied previously in the setting of ET blockade. However, changes in myocardial perfusion from endothelial dysfunction have been previously demonstrated in 20 subjects undergoing invasive coronary physiology evaluation.^15^ Technetium-99m was injected into the coronary artery immediately before the highest dose of the endothelium-dependent vasodilator acetylcholine. Perfusion defects were noted on imaging in all 7 subjects with a decrease in coronary blood flow, compared to 0 subjects whose flow increased. Even using a more relaxed cutoff of ≤50% increase in coronary blood flow, 7 of 11 subjects showed a perfusion defect compared to 0 of 9 subjects without endothelial dysfunction. Therefore, our findings of darusentan-induced changes in myocardial perfusion have precedent in this model using intracoronary delivery of acetylcholine.

Invasive coronary physiology was assessed at baseline and after 6 months in 47 subjects randomized to daily placebo vs an ET antagonist.^16^ While baseline flow increased by 15.3 cc/minute in treated subjects compared to 5.6 cc/minute in those taking placebo, this trend did not reach statistical significance (*P* = .25). However, it mirrors the increase in rest flow seen in our study while taking darusentan. Long-term ET antagonism significantly increased endothelium-dependent flow using acetylcholine (39.7% with blockade compared to −2.2% with placebo, *P* < .001) but not endothelium-independent CFR using adenosine (−0.3 in both groups, *P* = .65). Our study antagonized ET receptors for a much shorter period of time (2 weeks compared to 6 months) and did not differentiate between endothelium-dependent and endothelium-independent pathways.

A study of 19 community volunteers measured rest and hyperemic blood flow using cardiac PET before and during treatment with a selective ET type A antagonist.^17^ ET blockade did not alter the PRP, but increased resting blood flow from 0.63 to 0.85 cc/minute/g. However, no change in hyperemic blood flow was noted during adenosine infusion. These results are similar to our findings in Table 2 that ET antagonism increases resting blood flow without increasing the PRP, but hyperemic flows are not changed.

Prior work has studied the anti-hypertensive effects of darusentan in refractory hypertension. Our observed mean decrease in blood pressure of 9/7 mm Hg (systolic/diastolic) after 2 weeks (*P* < .001 compared to baseline) mirrors observations with the same dose of darusentan after 14 weeks in 81 patients with refractory hypertension of 9/5 mm Hg when offset by the placebo group.^13^ However, our observed mean increase in resting heart rate of 4 ± 7 bpm (*P* = .021) was not seen in that study. A mild decrease in hemoglobin due to hemodilution has been reported in other studies of ET antagonists,^13^,^16^ although our final labs were performed at least 10 days after the last dose of darusentan.

### Limitations

For pragmatic reasons we changed the enrollment HI cutoff value during study recruitment. However, relaxing the HI cutoff to include more homogeneous values would, if anything, tend to reduce the observed effect size compared to including more heterogeneous values as originally intended. Since 19 of the 20 subjects were male, our results apply essentially to that gender.

We did not measure serum levels of ET at baseline or during darusentan treatment. Our motivation for not doing so was the unclear relation between intracoronary effects and venous levels of ET. However, variations in baseline ET might explain the differential response to darusentan seen in Figure 3. For example, a subject with low levels of ET might not be expected to respond to its antagonism, as seen in several subjects with flat HI values between baseline and active treatment. ET levels might have provided a mechanistic explanation for varying treatment response. However, prior work in volunteers did not find an association between circulating ET-1 levels and changed rest flow with ET antagonism.^17^

## Conclusion

Homogeneity of resting myocardial perfusion measured by cardiac PET improved when taking the ET antagonist darusentan. A potential mechanism for improved homogeneity is increased and more uniform rest flow despite a slightly lower PRP. Our translational results support a mechanistic role for ET in regulating perfusion homogeneity in the human coronary circulation.
